# Involvement of 5-HT_1A_ receptors of the thalamic descending pathway in the analgesic effect of intramuscular heating-needle stimulation in a rat model of lumbar disc herniation

**DOI:** 10.3389/fnins.2023.1222286

**Published:** 2023-07-18

**Authors:** Yuhong Ma, Yijun Zhan, Jian Pei, Gang Ye, Yaoxin Chen, Wenyan Zhu, Haiyue Shen

**Affiliations:** ^1^Department of Acupuncture, Longhua Hospital, Shanghai University of Traditional Chinese Medicine, Shanghai, China; ^2^Department of Acupuncture and Traumatology, Shanghai Baoshan Luodian Hospital, Shanghai, China; ^3^Department of Rehabilitation Medicine, Tongji Hospital Affiliated to Tongji University, Shanghai, China

**Keywords:** intramuscular heating-needle stimulation, 5-HT1A receptor, descending modulation, lumbar disc herniation, thalamic ventromedial nucleus

## Abstract

**Background:**

Intramuscular (IM) heating-needle therapy, a non-painful thermal therapy, has been found to exert an analgesic effect *via* the thalamic ventromedial (VM) nucleus, solely by reducing the triggering threshold for descending inhibition; this could be modulated by intracephalic 5-hydroxytryptamine-1A (5-HT_1A_) receptors, rather than via the regular analgesia pathway. In this study, the effect and the potential serotonergic mechanism of IM heating-needle stimulation at 43°C were explored in the case of the pathological state of lumbar disc herniation (LDH).

**Methods:**

A modified classic rat model of LDH, induced via autologous nucleus pulposus implantation, was utilized. IM inner heating-needles were applied at the attachment point of skeletal muscle on both sides of the L4 and L5 spinous processes. WAY-100635 and 8-OH-DAPT, 5-HT_1A_ receptor antagonist and agonist, were separately injected into the bilateral thalamic mediodorsal (MD) and VM nucleus via an intrathalamic catheter. Nociception was assessed by bilateral paw withdrawal reflexes elicited by noxious mechanical and heat stimulation.

**Results:**

IM heating-needle stimulation at a temperature of 43°C for 30 or 45 min significantly relieved both mechanical and heat hyperalgesia in the rat model of LDH (*P* < 0.05). Heat hyperalgesia was found to be significantly enhanced by administration of WAY-100635 into the thalamic VM nucleus, blocking the effect of heating-needle stimulation in a dose-dependent manner (*P* < 0.05), while no effects were detected after injection into the thalamic MD nucleus (*P* > 0.05). Injection of 8-OH-DAPT into the thalamic MD nucleus exerted no modulating effects on either mechanical or heat hyperalgesia (*P* > 0.05).

**Conclusion:**

IM heating-needle stimulation at 43°C for 30 min may activate 5-HT_1A_ mechanisms, via the thalamic VM nucleus, to attenuate hyperalgesia in a rat model of LDH. This innocuous form of thermal stimulation is speculated to selectively activate the descending inhibition mediated by the thalamic VM nucleus, exerting an analgesic effect, without the involvement of descending facilitation of the thalamic MD nucleus.

## Introduction

Recent reports indicate that lower back pain is the fourth leading cause of disability-adjusted life-years (DALYs) among young adults aged 25 to 49 years (GBD, 2019), with lumbar disc herniation (LDH) being the most prevalent cause (Benzakour et al., 2019). The American College of Physicians strongly advocates for the prioritization of non-pharmacological therapies over pharmacological interventions for acute, sub-acute, and chronic lower back pain (Vijan et al., 2017).

There is increasing focus on the descending pain modulatory system as one of the various analgesic mechanisms that can be exploited for non-pharmacological treatment of LDH. This system is believed to be crucial in pain management (Yu et al., 2020). Previous research has identified two circuits that are specifically involved in discriminating and endogenously modulating emotional responses associated with pain: the thalamic mediodorsal (MD) nucleus–cingulate cortex–dorsolateral periaqueductal gray (PAG) circuit, and the thalamic ventromedial (VM) nucleus–insular cortex–ventrolateral PAG circuit (You et al., 2012; Lei and You, 2013; Lei et al., 2014; Xiao et al., 2015). Significantly, previous findings have demonstrated the crucial functions of the thalamic MD and VM nuclei in the regulation of descending facilitation and inhibition of mechanical and heat-induced nociception (You et al., 2012).

In the realm of clinical pain management, intramuscular (IM) heating-needle therapy at a temperature of ~43°C has been established as a non-painful thermal therapy with promising results (Lei et al., 2017). Previous experimentation has demonstrated that this particular form of stimulation operates solely on the VM nucleus to decrease the triggering threshold of descending inhibition, a process that can be regulated by intracephalic 5-HT1A receptors. This differs from the typical analgesia pathway, which involves the reversal of both descending inhibition and facilitation thresholds, as observed in IM heating-needle therapy at 46°C (You et al., 2014).

The efficacy of IM heating-needle stimulation in different pain models and the potential for development of more efficacious treatments for pain in pathological conditions remain to be explored. In this study, a modified classic LDH rat model was employed to examine the impact and potential serotonergic mechanism of IM heating-needle stimulation at 43°C in the pathological state of LDH.

## Materials and methods

### Animals

Male Sprague Dawley rats weighing 220–270 g at 10 weeks old were provided by the Animal Center of the College of Medicine, Xi'an Jiaotong University, and maintained in pairs in plastic boxes under a 12:12 h light/dark cycle (lights on at 08:00 a.m.) at 22–26°C with food and water available *ad libitum*. The animal experiments were approved by the Animal Care Committee of Shanghai University of Traditional Chinese Medicine in accordance with the National Institute of Health Guide for the Care and Use of Laboratory Animals (NIH Publications No. 80-23), as revised in 1996. Five days before the experiment, rats were placed in the experimental observation box for at least 1 h to adapt to the experimental environment.

### Rat model of non-compressive lumbar disc herniation

A modified classic LDH rat model of non-compressive LDH induced by autologous nucleus pulposus (NP) implantation was applied under anesthesia in the form of sodium pentobarbital (50 mg/kg, intraperitoneally) (Cho et al., 2016; Huang et al., 2017; Tang et al., 2022). The paraspinal muscles were dissected to expose the transverse processes of the L4–L5 segments. A hemilaminectomy was then performed to expose the left L4 nerve roots by removing the facet joint. Autogenous NP was harvested from the coccygeal discs and implanted into the L4 nerve roots without compression. Rats in the sham group underwent the same dissection and harvesting processes (tail transection), but no autologous NP implantation.

### IM heating-needle stimulation

The LDH model rats were anesthetized 14 days after induction of the model in the manner described above. With the rats fixed in a prone position, four internal heating needles (1.05 mm in diameter; 30 mm in length) were inserted into the periosteum. An internal heating therapeutic device (Model: NWX-1, Acuceuticals Co., Ltd, Shanghai, China) was connected and feedback control was provided. The internal heating needle was filled with the heating element and temperature probe for precise detection of changes in temperature (±0.25°C) (Lei et al., 2020). The temperature for heating-needle stimulation was set at 43°C, for 15, 30, or 45 min (You et al., 2014).

### Needle stimulation

Needle stimulation was performed using the same procedure as described above, except for heating. Stimulation was maintained for 45 min, corresponding to the maximum duration of stimulation in the IM heating-needle condition.

### Intracerebral microinjection with 5-HT_1*A*_ receptor antagonists and agonists

An intracerebral (IC) cannulation operation was carried out 7 days after induction of the model. Guide cannulas (OD 0.35 mm; ID 0.25 mm; RWD Life Science Co., Shenzhen, China) were advanced stereotaxically into the target nucleus of the thalamus. For the MD nucleus, the location was at -(2.3–2.8) mm from bregma, mediolateral 0.75 mm, and 5.2–5.4 mm in depth. For the VM nucleus, the location was at –(2.3–2.8) mm from the bregma, 1.2–1.5 mm mediolaterally, at a depth of 7.1–7.2 mm (Jiang et al., 2020). Cannula placement was carried out by an experienced, skilled researcher on our team, and the locations were guided by histological evidence from our previous work (Supplementary Figure 1). After catheterization, the rats were returned for 7 days of recovery, during which their behavior and motor function were monitored. Animals observed to have severe permanent neurological impairment or motor dysfunction were eliminated from the experiment.

One day after IM heating-needle stimulation for 30 min, 0.5 μl saline with 5-HT_1A_ receptor antagonist (WAY-100635) or agonist (8-OH-DPAT) was injected through the intrathalamic catheter using a 1 μl microsyringe with the rat fixed in place. The antagonist and agonist were commercially obtained from Sigma-Aldrich Chemie Gmbh (Germany) and were freshly prepared and dissolved in 0.9% saline. In brief, in the antagonist condition, doses of 0.5 nmol, 1 nmol, and 2 nmol of WAY-100635 were injected into the bilateral MD and VM nucleus. In the agonist condition, 1 nmol 8-OH-DPAT was injected into the bilateral MD nucleus. In a control condition, saline was injected into the bilateral MD nucleus and VM nucleus. The agents were infused at a constant speed within 30 s. The effects were assessed once per hour for the following 4 h (You et al., 2016; Jiang et al., 2020).

### Experimental design

The rats were randomly allocated to different experimental groups, with each group consisting of 10 rats, which was generally consistent with our previous studies. The assessor- and analyst-blinding method was applied in this experiment.

### Measurement of mechanical and heat sensitivity

Sensitivity to noxious mechanical stimulation was evaluated using the von Frey filament test. A hand-held electronic von Frey device (2290 Electrovonfrey^®^, IITC, Woodland Hills, CA, USA) with a rigid filament was used to detect the mechanical paw withdrawal threshold. The filament was applied to the heels of the bilateral hind paw to detect the foot withdrawal response. Sensitivity was recorded as paw withdrawal mechanical threshold (PWMT) in grams.

Sensitivity to noxious heat was assessed using the Hargreaves test, with a 390G Plantar Stimulator Analgesia Meter (IITC, Woodland Hills, CA). A high-intensity beam of radiant heat stimulus was aimed at the heels of the bilateral hind paw to determine the rat's tolerance to heat injury. The cut-off time for heat stimulation was set at 20 s to avoid any tissue damage. Sensitivity was recorded as paw withdrawal thermal latency (PWTL) in seconds.

Both of these measurements were performed 7, 10, and 14 days after induction of the model, and 2 h, 4 h, 1 day, 3 days, 5 days, and 7 days after needle stimulation. In the groups that received microinjections, the measurements were performed 30 min and 1 day after needle stimulation, and 1 h, 2 h, 3 h, and 4 h after microinjection. At every time point, measurements were taken three times with at least a 30-s interval.

### Statistical analysis

Statistical analysis was performed using SigmaStat (Systat Software Inc., San Jose, CA, USA); differences among groups over the entire observation period were tested by one-way ANOVA with *post hoc* Bonferroni tests. Data are expressed in the form mean ± standard error of the mean. When the data were non-parametric, as in the case of rotating pole test scores, a Kruskal–Wallis test was conducted. A *P*-value of < 0.05 was considered to represent statistical significance.

## Results

### Effects of needle stimulation on nociceptive paw withdrawal reflexes in normal rats

**Figure 1:**
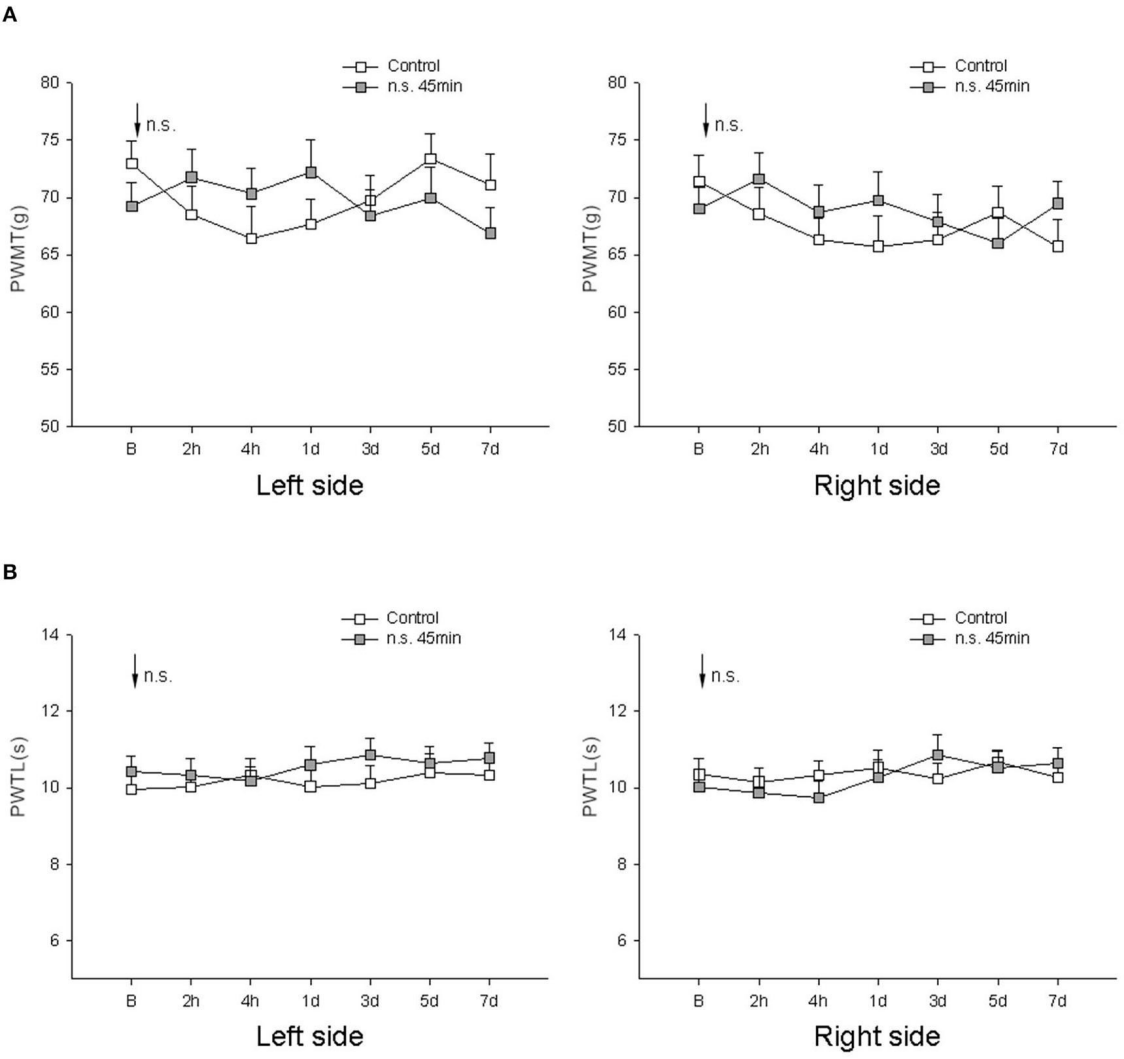
Comparison of bilateral PWMT and PWTL pre- and post-needle stimulation in the normal rats (*n* = 10/group). **(A)** Mechanical responses; **(B)** heat responses. Control, normal rats, receiving no treatment; n.s., needle stimulation group; B, baseline response before needle stimulation.

In comparisons with the control group, there was no significant effect of needle stimulation on bilateral PWMT or PWTL at 2 h, 4 h, 1 day, 3 days, 5 days, or 7 days after 45 min of needle stimulation (*P* > 0.05, one-way ANOVA; Figure 1).

### Effects of IM heating-needle stimulation on nociceptive paw withdrawal reflexes in rats after sham surgery

In this experiment, changes in PWMT and PWTL were first detected 7, 10, and 14 days after tail transection. The results showed that bilateral PWMT was significantly reduced (*P* < 0.05, one-way ANOVA) and bilateral PWTL was significantly prolonged (*P* < 0.01, one-way ANOVA) 7 days after tail transection; however, there were no significant differences in bilateral PWMT or PWTL at 10 days and 14 days after tail transection. It was observed that the rats remained stable and partially recovered 10–14 days after tail transection.

**Figure 2:**
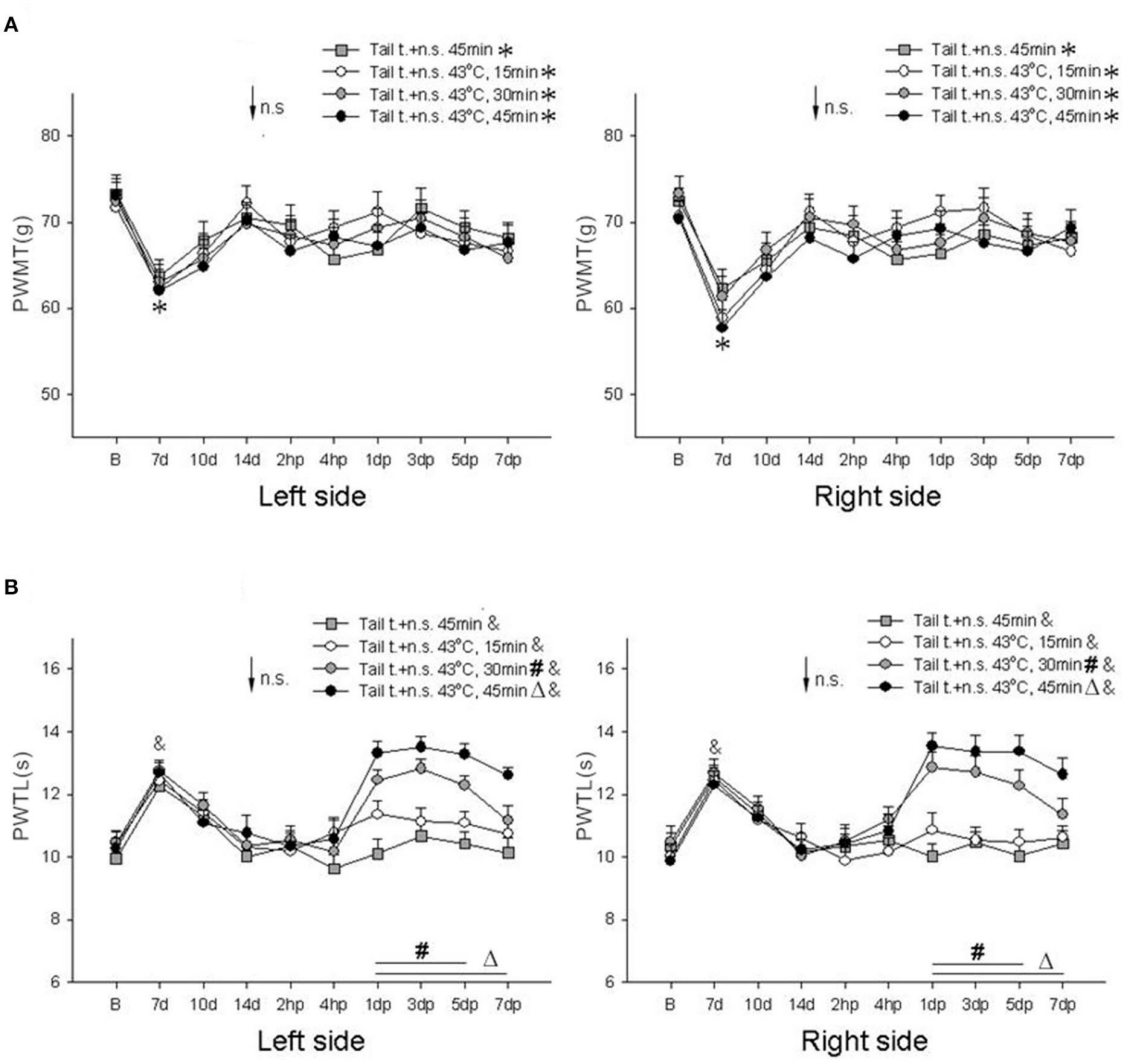
Comparison of bilateral PWMT and PWTL pre- and post-intervention in the sham surgery (tail transection) group of rats (*n* = 10/group). **(A)** Mechanical responses; **(B)** heat responses. Within-group comparisons, pre- vs. post-tail transection: *P < 0.05, ^&^*P* < 0.01. Within-group comparisons, pre- vs. post-intervention: ^#^*P* < 0.05 compared with 1–5 day(s), ^Δ^*P* < 0.05 compared with 1–7 day(s). Tail t, tail transection; B, baseline response before tail transection; n.s., needle stimulation.

Therefore, on the 14th day, the rats underwent interventions consisting of needle stimulation for 45 min, or IM heating-needle stimulation for 15 min, 30 min, or 45 min. There were no significant differences in bilateral PWMT 2 h, 4 h, 1 day, 3 days, 5 days, and 7 days after IM heating-needle stimulation in any of the groups (*P* > 0.05, one-way ANOVA; Figure 2A). Moreover, there was no significant difference in bilateral PWTL after needle stimulation for 45 min as compared to IM heating-needle stimulation for 15 min (*P* > 0.05, one-way ANOVA; Figure 2B). However, significantly prolonged bilateral PWTL was detected 1–5 day(s) after IM heating-needle stimulation for 30 min and 1–7 day(s) after IM heating-needle stimulation for 45 min (*P* < 0.05, one-way ANOVA; Figure 2B).

### Effects of IM heating-needle stimulation on nociceptive paw withdrawal reflexes in rats post-LDH surgery

In this experiment, the rats underwent interventions consisting of needle stimulation for 45 min, or IM heating-needle stimulation for 15 min, 30 min, or 45 min, 14 days after induction of the LDH model.

**Figure 3:**
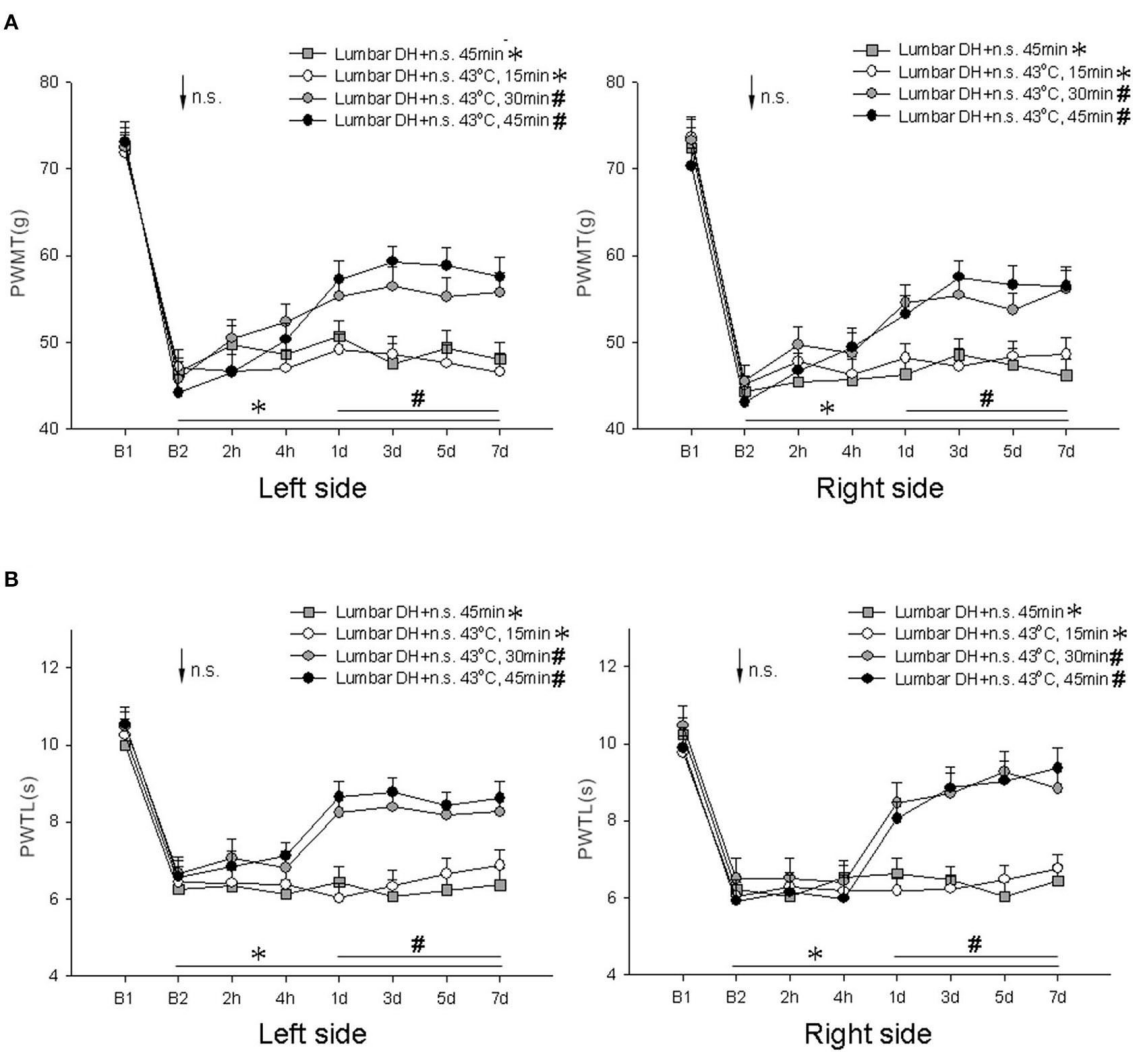
Comparison of bilateral PWMT and PWTL pre- and post-intervention in the LDH model group of rats (*n* = 10/group). Pre- vs. post-surgery comparison: *P < 0.01. **(A)** Mechanical responses; **(B)** heat responses. Pre- vs. post-intervention comparison: ^#^*P* < 0.05. Lumbar DH, lumbar disc herniation; B1, baseline response before induction of the model; B2, baseline response before needle stimulation, 14 days after induction of the model; n.s., needle stimulation.

Compared with pre-surgery results, bilateral PWMT was significantly decreased (*P* < 0.01, one-way ANOVA; Figure 3A), and bilateral PWTL was significantly reduced (*P* < 0.01, one-way ANOVA; Figure 3B) 14 days after induction of the model and at 2 h, 4 h, 1 day, 3 days, 5 days, and 7 days after various interventions.

Compared with the results prior to intervention but after induction of the model, bilateral PWMT and bilateral PWTL were not significantly different at 2 h, 4 h, 1 day, 3 days, 5 days, and 7 days after needle stimulation for 45 min or IM heating-needle stimulation for 15 min (*P* > 0.05, one-way ANOVA; Figure 3). However, a significant increase in bilateral PWMT and a prolonged PWTL were observed 1–7 day(s) after IM heating-needle stimulation for 30 min or 45 min (*P* < 0.05, one-way ANOVA; Figure 3).

### Effect of microinjection of 5-HT_1*A*_ receptor antagonist WAY-100635 into the thalamic VM nucleus on nociceptive paw withdrawal reflexes in rats post-LDH surgery under IM heating-needle stimulation

In this experiment, IM heating-needle stimulation was performed 14 days after induction of the model, and microinjection of 0.9% saline and 5-HT_1A_ receptor antagonist WAY-100635 at various concentrations into the thalamic VM nucleus was carried out 1 day after needle stimulation.

**Figure 4:**
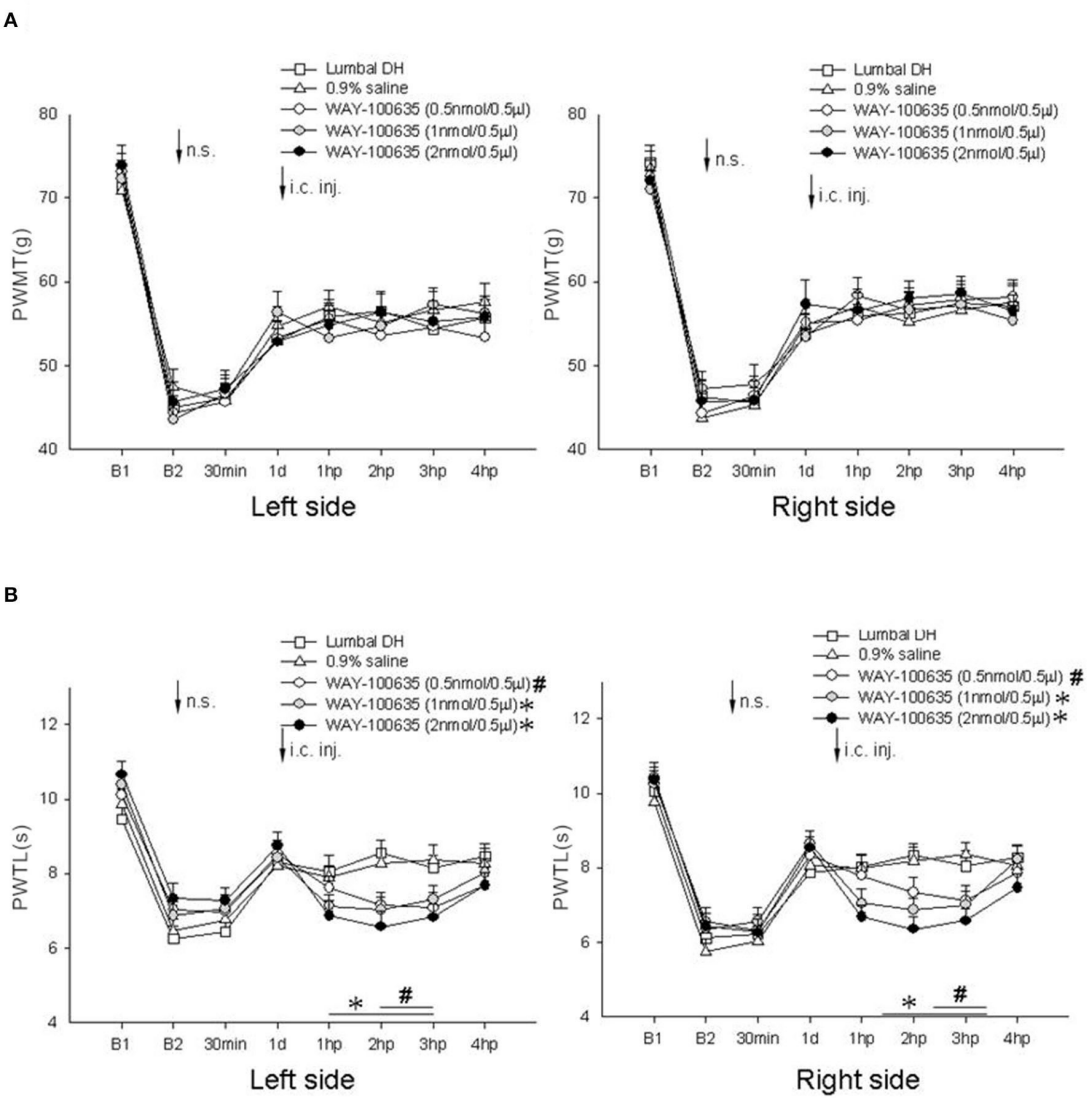
Effect of microinjection of WAY-100635 at various concentrations into the thalamic VM nucleus on bilateral PWMT and PWTL induced following needle stimulation in rats post-LDH surgery (*n* = 10/group). **(A)** Mechanical responses; **(B)** heat responses. Pre- vs. post-microinjection comparison: ^#^*P* < 0.05, *P < 0.05. B1, baseline response before induction of the model; B2, baseline response before needle stimulation, 14 days after induction of the model; n.s., needle stimulation.

No significant change in bilateral PWMT was detected after needle stimulation or 1–4 h after microinjection in any of the groups (*P* > 0.05, one-way ANOVA; Figure 4A).

For bilateral PWTL, no significant change was detected after needle stimulation or 1–4 h after VM microinjection of 0.9% saline (*P* > 0.05, one-way ANOVA; Figure 4B). However, a clear reduction of bilateral PWTL was observed 2–3 h after VM microinjection of 0.5 nmol WAY-100635, as well as 1–3 h after VM microinjection of 1 nmol or 2 nmol WAY-100635 (*P* < 0.05, one-way ANOVA; Figure 4B).

### Effect of microinjection of 5-HT_1*A*_ receptor antagonist WAY-100635 into the thalamic MD nucleus on nociceptive paw withdrawal reflexes in rats post-LDH surgery under IM heating-needle stimulation

In this experiment, microinjection of 0.9% saline and 5-HT_1A_ receptor antagonist WAY-100635 at various concentrations into the thalamic MD nucleus was carried out.

**Figure 5:**
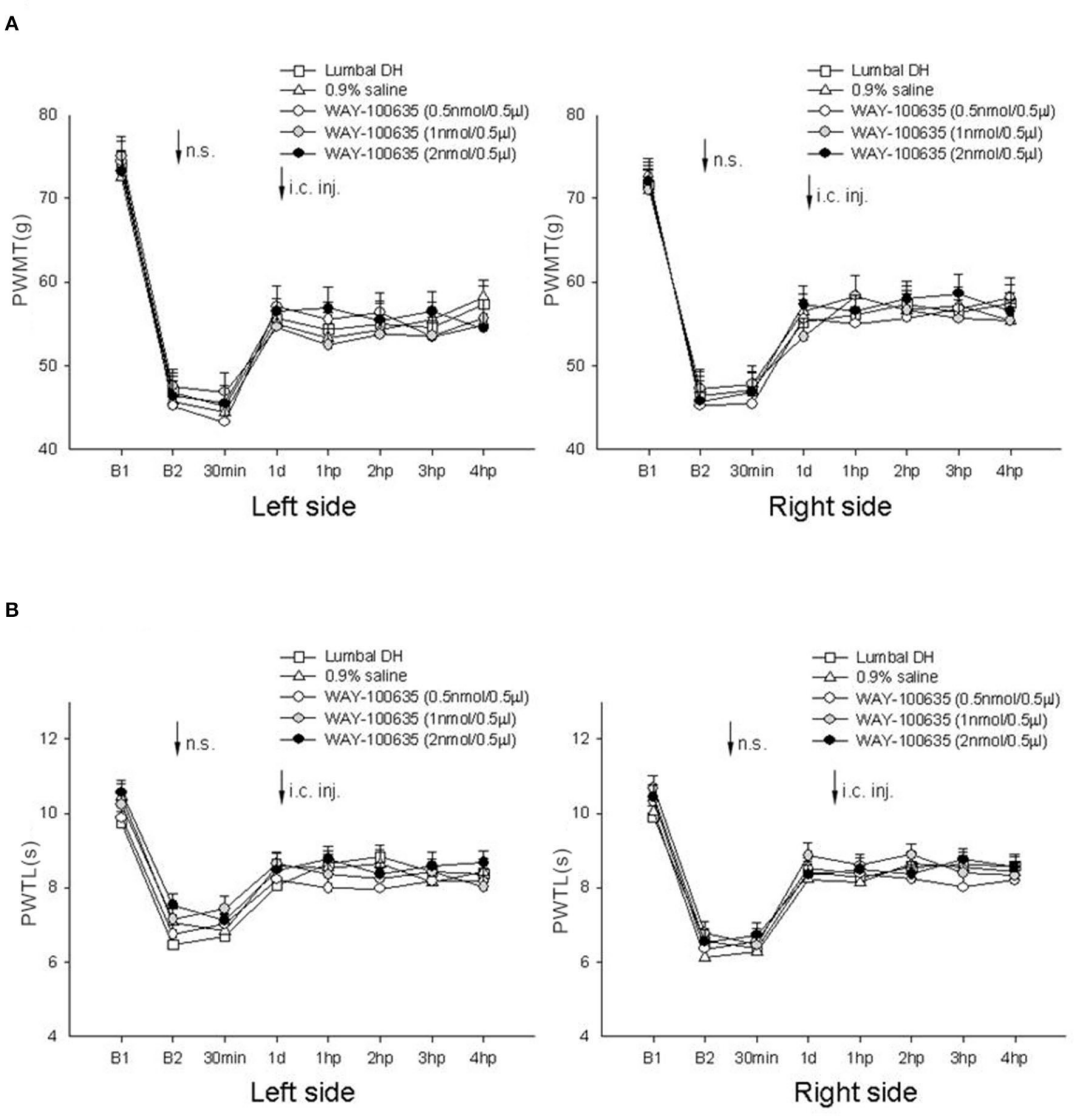
Effect of microinjection of WAY-100635 at various concentrations into the thalamic MD nucleus on bilateral PWMT and PWTL induced following needle stimulation in rats post-LDH surgery (*n* = 10/group). **(A)** Mechanical responses; **(B)** heat responses. B1, baseline response before induction of the model; B2, baseline response before needle stimulation, 14 days after induction of the model; n.s., needle stimulation.

No significant difference in bilateral PWMT or PWTL was detected after needle stimulation or 1–4 h after microinjection in any of the groups (*P* > 0.05, one-way ANOVA; Figure 5).

### Effect of microinjection of 5-HT_1*A*_ receptor agonist 8-OH-DPAT into the thalamic MD nucleus on nociceptive paw withdrawal reflexes in rats post-LDH surgery under IM heating-needle stimulation

In this experiment, microinjection of 0.9% saline and 5-HT_1A_ receptor agonist 8-OH-DPAT at various concentrations into the thalamic MD nucleus was carried out.

**Figure 6:**
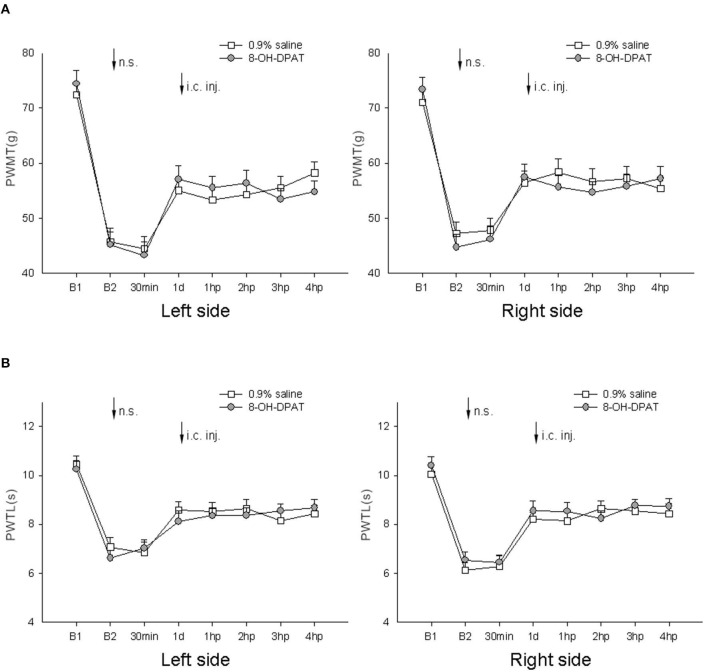
Effect of microinjection of 8-OH-DPAT at various concentrations into the thalamic MD nucleus on bilateral PWMT and PWTL induced following needle stimulation in rats post-LDH surgery (*n* = 10/group). **(A)** Mechanical responses; **(B)** heat responses. B1, baseline response before induction of the model; B2, baseline response before needle stimulation, 14 days after induction of the model; n.s., needle stimulation.

No significant difference in bilateral PWMT or PWTL was detected after needle stimulation or 1–4 h after microinjection in any of the groups (*P* > 0.05, one-way ANOVA; Figure 6).

## Discussion

IM heating-needle stimulation provides a constant adjustable temperature at the top point of the needle because the instrument consists of a concentric needle and a connected therapeutic inner heating device. Previous studies have documented evidence that IM heating-needle stimulation at 43°C could induce positive effects in arthrogenic pain and myofascial pain syndrome in both humans and animal models (Lei et al., 2017, 2020; Ma et al., 2021b); these findings are also supported by a meta-analysis including 17 studies with 1,539 LDH patients (Wang et al., 2016). Experiments have revealed the physiological mechanism of activation of the thalamic VM nucleus (Wang et al., 2016), which could be modulated by intracephalic 5-HT_1A_ receptors.

This study explored the effect of IM heating-needle stimulation in a rat model of LDH, with the aim of investigating the analgesic mechanism of this treatment in pathological conditions. The results showed a promising effect of IM heating-needle stimulation at 43°C in the rat LDH models in terms of relief of both heat and mechanical hyperalgesia. The evidence suggested that the most effective and efficient treatment duration was 30 min, which was consistent with the findings of our previous research (You et al., 2014; Lei et al., 2020). Since this study was our first attempt at conducting this intervention for the pathological state of LDH, we found that the treatment took longer to take effect (on average 4 h) than previous studies in which we examined other physiological states. Another interesting finding, which differed from the general consensus, was that our sham surgery animals exhibited significant heat and mechanical hyperalgesia 7 days after the operation. This phenomenon was due to the fact that the sham surgery animals also underwent hemilaminectomy and the removal of the facet joint, indicating the occurrence of nerve damage; this is in line with the theory of latent sensitivity, under which the hyperalgesic phase after nerve damage is a rather complicated condition, lasting for as long as 30 days, including masked hyperalgesia in the remission phase (Marvizon et al., 2015). Moreover, the findings on microinjection of 5-HT_1A_ antagonist and agonist into the VM and MD nucleus, respectively, provided a potential direction regarding the serotonergic mechanism of IM heating-needle stimulation in thalamic VM descending inhibitory modulation.

The role of the thalamic VM and MD nuclei in discrimination and descending control of nociception was elucidated through a series of experiments. Specifically, the thalamic VM nucleus-related circuit could be activated by both innocuous and noxious heat stimuli, which exerts a descending inhibitory effect on thermal nociception (You et al., 2022). In contrast, the thalamic MD nucleus is involved in distinguishing ascending signals evoked by noxious mechanical stimulation only, and exerts a descending facilitatory effect on mechanical hyperalgesia, rather than heat hypersensitivity. In view of the characteristic ways in which innocuous heat stimuli can be evoked, the thalamic VM nucleus is increasingly the focus in development of novel treatment regimens for pathological pain conditions.

5-HT is widely acknowledged to be involved in human pain signaling via ascending and descending modulatory pathways (Martin et al., 2017), and endogenous 5-HT is active in the descending inhibition of nociception rather than the descending facilitation of pain (You et al., 2010). In this study, 5-HT_1A_ was selected to investigate the serotonergic mechanism of IM heating-needle stimulation, which has previously been recognized to involve the activity of the descending modulatory pathway (Silveira et al., 2010). In view of our previous conclusion that innocuous stimulation triggers the VM nucleus only, in this study, we microinjected 5-HT_1A_ receptor antagonist at different levels of concentration into the VM and MD nuclei, and 5-HT_1A_ receptor agonist into the MD nucleus only, in order to investigate their functions. It was observed that microinjection of 5-HT_1A_ receptor antagonist into the thalamic VM nucleus could block heat hypoalgesia induced by IM heating-needle stimulation in a dose-dependent manner, while microinjection into the thalamic MD nucleus exerted no effects on the results of IM heating-needle stimulation, whether 5-HT_1A_ receptor antagonist or agonist was injected. These results confirmed the role of the thalamic VM nucleus in endogenous descending inhibition of pain induced by innocuous thermal stimulation, as well as the role of the thalamic MD nucleus in endogenous descending facilitation of pain caused by nociceptive mechanical stimulation (You et al., 2012). A similar conclusion was drawn based on evidence on the analgesic effects of pulsed radiofrequency (PRF) therapy at 37°C and 42°C in a rat model of inflammatory pain, which could be inhibited by the intrathecal injection of serotonergic receptor antagonist (Hagiwara et al., 2008).

For further investigation of the serotonergic mechanism of IM heating-needle stimulation, methods for visualization of serotonin transporters (SERTs) in animal brains, such as 4-[18F]-ADAM/PET imaging, could be applied, since such tools have been utilized to examine the modulation of SERT density by PRF (Ma et al., 2021a), a promising non-pharmacological therapy for chronic pain.

## Conclusion

The analgesic effects of IM heating-needle stimulation at 43°C in a rat model of LDH were found to lie in the 5-HT_1A_ mechanisms of the thalamic VM nucleus. We hypothesize that innocuous thermal stimulation might selectively activate descending inhibition mediated by the thalamic VM nucleus, rather than involving descending facilitation by the thalamic MD nucleus.

## Data availability statement

The raw data supporting the conclusions of this article will be made available by the authors, without undue reservation.

## Ethics statement

The animal study was reviewed and approved by the Animal Care Committee of Shanghai University of Traditional Chinese Medicine.

## Author contributions

JP, YM, and GY designed the study. YM, YZ, YC, WZ, and HS conducted the experiments and data analysis. YM and YZ completed the drafting and editing of the manuscript. All authors contributed to the article and approved the submitted version.

## Supplementary material

Supplementary Figure 1 Histological basis of the location of the placement of cannulas into VM and MD.
