# Supplementary figures and images for: Involvement of 5-HT1A receptors of the thalamic descending pathway in the analgesic effect of intramuscular heating-needle stimulation in a rat model of lumbar disc herniation
Source: Front Neurosci. 2023 Jul 18;17:1222286. doi: 10.3389/fnins.2023.1222286 (PMC10390831; doi:10.3389/fnins.2023.1222286)

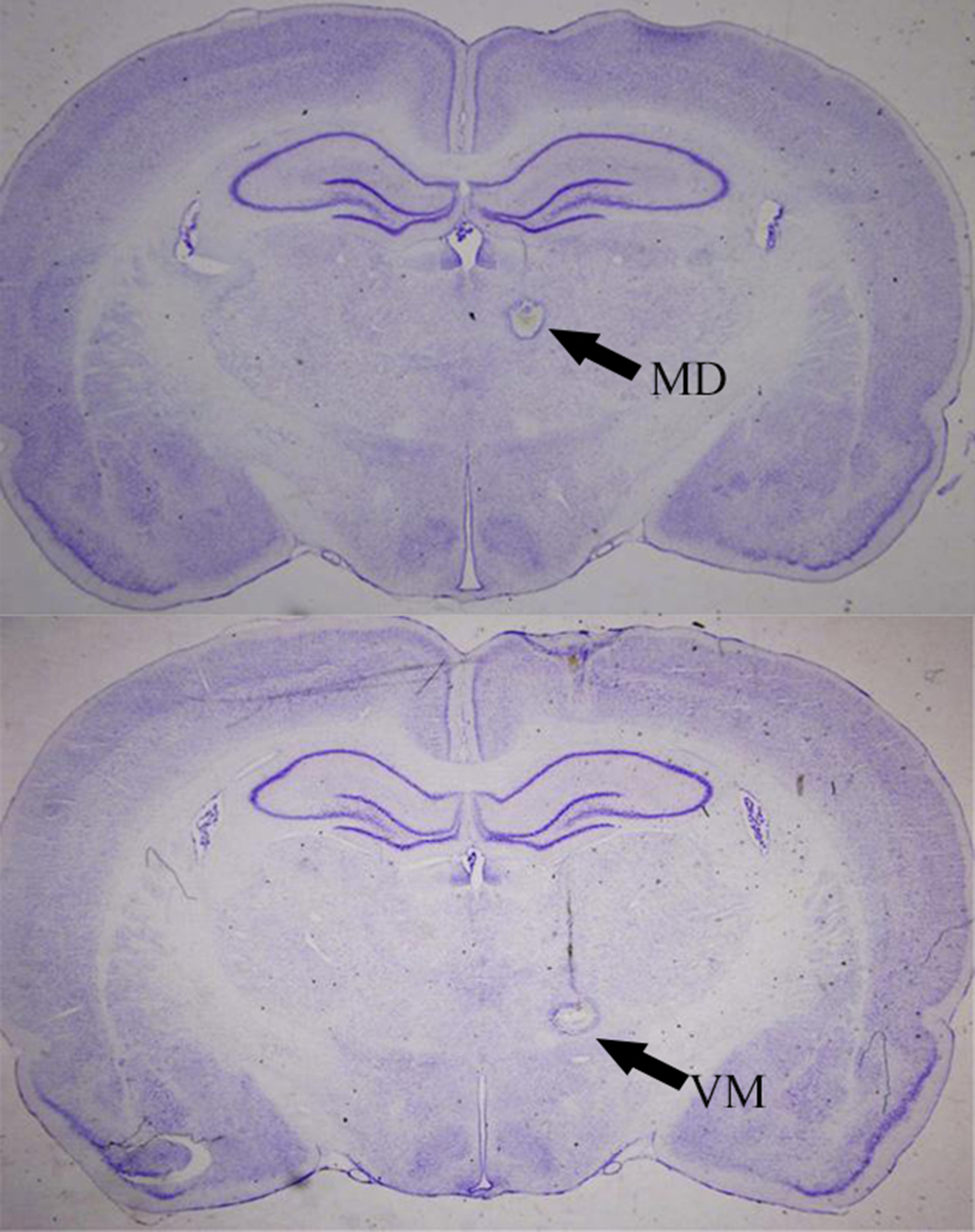

Supplement: Supplementary Figure 1 — Histological basis of the location of the placement of cannulas into VM and MD. [file Image_1.JPEG]
